# Haploinsufficiency of ZNF251 causes DNA-PKcs-dependent resistance to PARP inhibitors in BRCA1-mutated cancer cells

**DOI:** 10.21203/rs.3.rs-2688694/v1

**Published:** 2023-04-06

**Authors:** Huan Li, Srinivas Chatla, Xiaolei Liu, Umeshkumar Vekariya, Dongwook Kim, Matthew Walt, Zhaorui Lian, George Morton, Zijie Feng, Dan Yang, Hongjun Liu, Katherine Reed, Wayne Childers, Xiang Yu, Jozef Madzo, Kumaraswamy Naidu Chitrala, Tomasz Skorski, Jian Huang

**Affiliations:** Coriell Institue for Medical Research; Temple University; University of Pennsylavania School of Medecine; Lewis Katz School of Medicine; Coriell Institue for Medical Research; Coriell Institue for Medical Research; Coriell Institute for Medical Research; Temple University Lewis Katz School of Medicine; University of Pennsylavania School of Medecine; Coriell Institue for Medical Research; IPhase Parma Services LLC.; Inovio Pharmaceuticals; Shanghai Jiao Tong University; Coriell Institute; University of Houston; Temple University; Coriell Institue for Medical Research

## Abstract

Poly (ADP-ribose) polymerase (PARP) inhibitors represent a promising new class of agents that have demonstrated efficacy in treating various cancers, particularly those that carry *BRCA1/2* mutations. The cancer associated *BRCA1/2* mutations disrupt DNA double strand break (DSB) repair by homologous recombination (HR). PARP inhibitors (PARPis) have been applied to trigger synthetic lethality in *BRCA1/2*-mutated cancer cells by promoting the accumulation of toxic DSBs. Unfortunately, resistance to PARPis is common and can occur through multiple mechanisms, including the restoration of HR and/or the stabilization of replication forks. To gain a better understanding of the mechanisms underlying PARPi resistance, we conducted an unbiased CRISPR-pooled genome-wide library screen to identify new genes whose deficiency confers resistance to the PARPi olaparib. Our study revealed that ZNF251, a transcription factor, is a novel gene whose haploinsufficiency confers PARPi resistance in multiple breast and ovarian cancer lines harboring BRCA1 mutations. Mechanistically, we discovered that *ZNF251* haploinsufficiency leads to constitutive stimulation of DNA-PKcs-dependent non-homologous end joining (NHEJ) repair of DSBs and DNA-PKcs-mediated fork protection in *BRCA1*-mutated cancer cells (BRCA1mut + *ZNF251*KD). Moreover, we demonstrated that DNA-PKcs inhibitors can restore PARPi sensitivity in BRCA1mut + *ZNF251*KD cells *ex vivo* and *in vivo*. Our findings provide important insights into the mechanisms underlying PARPi resistance and highlight the unexpected role of DNA-PKcs in this phenomenon.

## Introduction

The poly (ADP-ribose) polymerases (PARPs) - also known as NAD + ADP-ribosyltransferases - are an emerging family of 18 enzymes that share the ability to catalyze the transfer of ADP-ribose to target proteins (poly ADPribosylation)^1,2^. PARPs play an important role in various cellular processes, including modulation of chromatin structure, transcription, replication, recombination, and DNA repair^3^. PARP1 is the most potent enzyme of this group and accounts for 80–90% of DNA damage-induced PARylation; it also plays a key role in DNA damage response (DDR), including the repair of DNA single-strand breaks (SSBs) and double-strand breaks (DSBs)^4–6^. SSBs are repaired by PARP1-mediated base-excision repair (BER). DSBs may be repaired by three classical pathways: BRCA1/2-dependent homologous recombination (HR), DNA-PKcs-mediated nonhomologous end joining (NHEJ), and PARP1-mediated alternative NHEJ (Alt-NHEJ). These DNA repair pathways can either work independently or coordinately to prevent/repair different types of DSBs.

Mutations in the *BRCA1/2* genes that result in dysfunctional HR incur a high risk of breast and ovarian cancer development. Both BRCA1 and BRCA2 interact with various proteins involved in the HR repair pathway and appear indispensable for this process, acting at different stages in DSB repair. Because PARP inhibitors (PARPis) induce DSBs in cells with dysfunctional HR, cells harboring *BRCA1/2* mutations are particularly sensitive to the treatments with PARPis^3,7^. Current FDA-approved PARPis: olaparib, rucaparib, niraparib, and talazoparib are NAD + competitors, thus blocking the poly (ADP ribose) polymerase activity. Failure to repair SSB lesions due to PARP1 inhibition generates toxic DSBs in cells displaying HR deficiency, resulting in synthetic lethality. Unfortunately, the majority of patients with *BRCA1/2* mutated tumors who initially show improvements after PARPis treatment develop resistance, which results in disease relapse and progression.

To investigate the mechanisms underlying resistance to PARP inhibitors (PARPis), we conducted a genome-wide CRISPR screen to identify gene mutations that confer resistance to olaparib. Our findings reveal that haploinsufficiency in zinc finger 251, resulting in partial knockdown of ZNF251 protein (referred to as *ZNF251*KD), causes resistance to olaparib in multiple *BRCA1*-mutated cancer cell lines. Moreover, we observed that breast cancer cells with *BRCA1* mutation and *ZNF251*KD (*BRCA1*mut + *ZNF251*KD) were not only resistant to various PARPis, but also to platinum-based drugs and DNA polymerase theta (Polθ) inhibitors.

Our study further demonstrated that the activation of the DNA-PKcs-mediated NHEJ repair pathway and DNA-PKcs-mediated replication fork stabilization are associated with olaparib resistance conferred by *ZNF251*KD in *BRCA1*-mutated cells. Critically, we also showed that *BRCA1*mut + *ZNF251*KD breast cancer cells were sensitive to DNA-PKcs inhibitors, which restored their sensitivity to PARPi *ex vivo* and *in vivo*. These results suggest that *ZNF257*KD-mediated resistance to PARPis involves the DNA-PKcs pathway, which may represent a therapeutic target for overcoming PARPi resistance in *BRCA1*mut + *ZNF251*KD breast cancer cells.

## Materials And Methods

### Cell lines and cell culture

MDA-MB-436 and HCC1937 were purchased from ATCC. MDA-MB-436 and Ovcar8 cells were maintained in DMEM supplemented with 10% FBS and 1% penicillin/streptomycin. HCC1937 cells were cultured in RPMI 1640 supplemented 10% FBS and 1% penicillin/streptomycin. All cell lines were analyzed and authenticated by morphologic inspection and biochemical examination of the *BRCA1* mutation pathway as well as short tandem repeat profiling analysis. Mycoplasma testing was also performed to exclude the possibility of mycoplasma contamination of all cell lines.

### Chemical Compounds

Olaparib (Catalog# A4154), cisplatin (Catalog# A8321), carboplatin (Catalog# A2171), and 5-fluorouracil (Catalog# A4071) were all purchased from APExBIO company. UPF 1096 (Catalog# S8038), NMS-P118(Catalog# S8363), stenoparib (E7449) (Catalog# S8419), niraparib (Catalog# S2741), rucaparib (Catalog# S4948), and veliparib (ABT-888) (Catalog# S1004) were all purchased from Selleckchem.

Cisplatin (Catalog# A10221) was purchased from AdooQ Bioscience. ART-558(Catalog# HY-141520), DNA-PK inhibitors PIK-75 hydrochloride (Catalog# HY-13281), Nedisertib (Catalog# HY-101570) and AZD-7648 (Catalog# HY-111783), RAD51 Inhibitor B02 (Catalog# HY-101462), and olaparib (for *in vivo* experiment: Catalog# HY-10162) were all purchased from MCE (MedChem Express). ART-812 was synthetized by Dr. Wayne Childers at Temple University School of Pharmacy. All compounds were dissolved, aliquoted, and stored following the manufacturer’s instructions.

### Pooled Genome-wide CRISPR/Cas9 Screen

The GeCKO CRISPR library was purchased from Addgene (#1000000048), amplified, and packaged as lentivirus based on the instructions on Addgene website. The CRISPR screen was performed as described previously^8^. In brief, MDA-MB-436 cells were transduced with lentivirus carrying GeCKO library, and puromycin selection was performed for 3 days. Then we treated transduced MDA-MB-436 cells with olaparib for 14 days, the medium was changed with adding fresh olaparib every three days during 14 days screen, and the surviving cells were harvested. The genomic DNA was extracted, and PCR was carried out before deep sequencing of sgRNA sequence in the surviving cells’ genome. All deep sequencing data are available at GEO (series accession number GSE205221). For data analysis, we calculated the enrichment score as: The enrichment score= (sgRNA number from the reads)/ (sgRNA number in the library) X log_2_ (average abundance). The sgRNAs used for validations were synthesized and constructed as described^8^. Primer sequences are shown in Supplementary Table S1.

### T7EN1 assays and DNA sequencing

The T7EN1 assay was performed as described previously^8^. To identify the *ZNF251* mutations, the purified PCR product was cloned into the pCR2.1-TOPO TA vector (TOPO TA cloning kit; Life Technologies) and sequenced by Sanger sequencing. The primers used for Sanger sequencing were listed in Supplementary Table S1.

### Generation of mutant single clones

500 transduced MDA-MB-436 cells were mixed with 1 ml of methylcellulose (MethoCult H4034 Optimum, Stem Cell Technologies) in a 6-well cell culture plate and cultured at 37°C in a 5% CO_2_ incubator. Two weeks later, single colonies were picked and cultured in a 96-well plate with the complete medium supplemented with 2% penicillin/ streptomycin. The cells were passaged every two or three days, and 1/3 of cells were collected for genomic DNA extraction. Then *ZNF251* target region was PCR amplified and sequenced.

### Cell viability assay

1×10^4^ cells were cultured with 100μl of complete medium in a 96-well plate and treated as indicated. Cell viability was measured at different time points as described with the trypan blue exclusion viability test. The final viable cell number was calculated based on the growth standard curve. All the key viability experiments were confirmed by the MTS assay (Promega, Catalog# G3582) and CCK-8 assay (APExBIO company, Catalog# K1018).

### Off-target effect examination

Off-target sites were predicted using an online search tool (http://crispr.mit.edu). 3bp mismatches compared with the target consensus sequence were allowed. The predicted off-target sequences were searched using UCSC browse, and 500bp flanking the sites were PCR-amplified in primary cells and single mutation clones. The PCR product was subjected to the T7EN1 assay to determine the mutation. The PCR product was then cloned into a TA vector and Sanger sequenced to identify mutations.

### ZNF251 complementation experiment

Exponentially growing MDA-MB-436 *ZNF251* WT and KD cells were seed in six-well plates (1 million cells/well) and transfected with pcDNA3.1 vector or human *ZNF251* on pcDNA3.1 plasmid carrying a neomycin resistance (neo) gene. After transfection with 1 μg and 2 μg plasmid respectively, the cells were selected with G418 (400 ug/ml) in the culture medium for 2 weeks to keep selecting neomycin resistant cells for generating stably transfected cell lines^9,10^. Then plated into 96-well plates at a density of 1× 10^4^ cells per well in triplicates. Next day, the transfected cells were treated with DMSO or olaparib for 3 days and cell viability was measured. Expression of *ZNF251 (ZNF251* mRNA) was measured by real-time PCR in control and human *ZNF251* on pcDNA3.1 plasmid transfected MDA-MB-436 cells. It was performed with iTaq^™^ Universal SYBR^®^ Green One-Step Kit (Bio-Rad cat#1725150). The expression level of *ZNF251* was normalized to housekeeping *GAPDH* gene.

### Immunoblot analysis

Nuclear and total cell lysates were obtained as described before^11^ and analyzed by SDS-PAGE using primary antibodies against: ATM (Santa Cruz Biotechnology #sc-135663), CtIP (Abcam #ab-70163), 53BP1 (Abcam #ab-175933), SLFN11 (Santa Cruz Biotechnology #sc-515071), BRCA1 (ThermoFisher Scientific #MA1–23164), BRCA2 (Santa Cruz Biotechnology #sc-28235), PALB2 (Proteintech #14340–1-AP), RAD51 (Abcam #ab-88572), RAD52 (Santa Cruz Biotechnology #sc-365341), RAD54 (Santa Cruz Biotechnology #sc-374598), DNA-PKcs (Bethyl #A300–518A), Ku70 (Santa Cruz Biotechnology #sc-17789), Ku80 (ThermoFisher Scientific #MA5–15873), DNA ligase 4 (ThermoFisher Scientific #PA5–40826), PARP1 (Santa Cruz Biotechnology #sc-74470), PARP2 (Santa Cruz Biotechnology #sc-393310), PARP3 (Santa Cruz Biotechnology #sc-390771), DNA ligase 3 (Santa Cruz Biotechnology #sc-135883), Polθ (MyBioSource #MBS9612322), lamin B (Abcam #ab-16048–100), and β-actin (Santa Cruz Biotechnology #sc-47778) and the following secondary antibodies conjugated to HRP (horseradish peroxidase): goat anti-rabbit (EMD Millipore #12–348) and goat anti-mouse (EMD Millipore #AP181P). ZNF251 western analysis was performed with ZNF251 antibody (Proteintech cat# 25601–1-AP) and GAPDH antibody (Cell signaling technology cat#2118). For quantification of western analysis, ImageJ software was used to measure the density of the protein bands.

### DNA damage/repair assays

DSBs were detected by neutral comet assay as described before^11^ with modifications. Briefly, comet assays were performed using the Oxiselect Comet Assay Kit (Cell Biolabs #STA-355) according to the manufacturer’s instructions. Images were acquired by an inverted Olympus IX70 fluorescence microscope using a FITC filter, and the percentage of tail DNA of individual cells was calculated using the OpenComet plugin of ImageJ. HR, D-NHEJ, and Alt-NHEJ were measured using DR-GFP (HR), EJ2-GFP (D-NHEJ), and EJ5-GFP (Alt-NHEJ) reporter cassettes as described before^11^. Briefly, the reporter plasmid was digested by I-SceI endonuclease, and the repaired GFP cells were counted by flow cytometer. The result was calculated by total restored GFP positive cells / total transfected M-cherry or BFP positive cells.

### Mice and in vivo studies

6–8 weeks-old female NOD/SCID/IL-2Rγ (NSG) mice (Jackson Laboratories) were injected subcutaneously with 1×10^6^ MBA-MD-436 cells in the flank. Mice were randomized to treatment groups when tumor sizes reached 50–60 mm^3^. For the first set of the experiments, all animals with wildtype or *ZNF251* KD tumors of 50–60 mm^3^ were randomized into two groups (n = 4/group), which were intraperitoneally treated with vehicle or olaparib (10mg/kg) daily for four weeks, respectively. For the second set of experiments, all mice were randomly divided into four groups and intraperitoneally injected daily with either vehicle, olaparib (10mg/kg), DNA-PK inhibitor PIK-75 (10mg/kg), or olaparib (10mg/kg) plus PIK-75(10mg/kg) for four weeks. Since the start of the experiment, tumor volumes (V) were measured every three days based on the formula V = LxW^2^×0.5, where L represents the largest tumor diameter and W represents the perpendicular tumor diameter^12^. After four weeks, all mice were euthanized and tumors were dissected out, imaged, weighed, or used for further characterization. All experiments involving animals were approved by the Cooper University and the IPhase Pharma Services LLC Institutional Animal Care and Use (IACUC) Committee.

### Fork protection assay/DNA fiber assay

At stalled forks, degradation of DNA fibers was assessed as follows. Exponentially growing MDA-MB 436 *ZNF251* WT and KD cells were treated with 5 uM Olaparib and/or 8 uM Plk-75 for 48 hrs. Cells were sequentially pulse-labeled with 50 μM of 5-chloro-2’-deoxyuridine thymidine (CldU) (Sigma-Aldrich) and 250 μM of idoxuridine (IdU) (Sigma-Aldrich) for exactly 30 min each, washed once with 1 × PBS, and treated with 4 mM HU for 4 hr. Cells were collected and resuspended in 1× PBS at a concentration of 500 cells/ul. 2.5 μl of cell suspension was diluted with 7.5 μl of lysis buffer (200 mM Tris-HCl pH 7.5, 50 mM EDTA, and 0.5% [w/v] SDS) on a glass slide and incubated for 8 min at RT. The slides were titled at 15–60°, air-dried, and fixed with 3:1 methanol/acetic acid for 10 min. Slides were denatured with 2.5 M HCl for 90 min, washed with 1 × PBS, and blocked with 2% BSA (Carl Roth) in PBS for 40 min. The newly replicated CldU and IdU tracks were labeled for 1.5 hr with anti-BrdU antibodies recognizing CldU (1:300, Abcam) and IdU (1:100, BD Biosciences), followed by 1 hr incubation with secondary antibodies anti-mouse Alexa Fluor 594 (1:500, #A11062, Life Technologies) and anti-rat Alexa Fluor 488 (1:500, #A21470, Life Technologies). The incubations were performed in the dark in a humidified chamber. After 5 washes in PBST for 3 min, mount coverslip with 20 ul mounting media. DNA fibers were visualized using a Leica SP8 Confocal microscope at a 63X objective magnification, and images were analyzed using ImageJ software.

### Bioinformatics analysis of ZNF251 expression in the cells sensitive and resistant to PARPi olaparib

To analyze the expression of *ZNF251* expression in the cells sensitive and resistant to PARPi olaparib, we performed bioinformatics analysis. Datasets for the respective inhibitors were downloaded from the Gene Expression Omnibus database (http://www.ncbi.nlm.nih.gov/geo), a large public repository for high-throughput molecular abundance data – specifically, gene expression data^13^. Dataset GSE165914 was utilized for the analysis of *ZNF251* expression in olaparib sensitive and resistant cells^14^. Statistical analysis was performed using the Graph Pad Prism 9.

### Quantification and Statistical Analysis

All statistical analyses were performed by GraphPad Prism 8. Cell viability data were analyzed by two-way ANOVA tests. The neural comet assay data was analyzed by Mann-Whitney Rank Sum Test. The data of DNA repair assay and *in vivo* experiments were analyzed by unpaired t-test with Welch’s correction.

### Data availability

The data generated in this study are available within the article and its supplementary data files. All deep sequencing data of our CRISPR screen are available at GEO (series accession number GSE205221).

## Results

### A genome-wide CRISPR screen identified ZNF251 as a critical factor regulating sensitivity of BRCA1-mutated cells to PARPis

**Figure 1.**
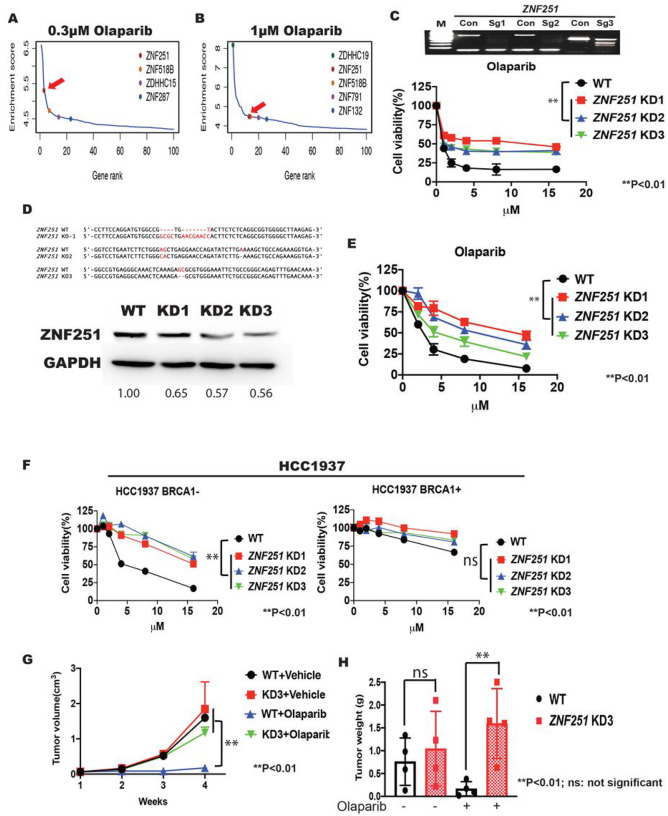
A genome-wide CRISPR screen identified *ZNF251* whose knockdonw caused Olaparib resistance in a breast cancer line A CRISPR screen in MDA-MB-436 *BRCA1*-mutated breast cancer cells uncovered *ZNF251* genes whose loss-of-function confers olaparib resistance. A, B. Enrichment of specific sgRNAs that target each gene after 14 days of olaparib treatment and identification of top candidate genes. The x axis represents enriched genes, and the y axis represents sgRNA enrichment score, which was calculated using (sgRNA number from the reads)/(sgRNA number in the library)/log_2_ (average abundance). Arrow indicates *ZNF251* gene. C. Top panel: T7EN1 assay analysis of specific sgRNA-mediated in/dels at *ZNF251* locus in MDA-MB-436 cells. Bottom panel: Cell growth curve of parental *(ZNF257* WT) and *ZNF251* KD MDA-MB-436 breast cancer cells following treatment with olaparib. The results represent three independent experiments. D. Top panel: Sanger sequencing data of three *ZNF257* KD clones. Bottom panel: Western blot analysis of wildtype (WT) and *ZNF251*KD clones 7–3 of MDA-MB-436 breast cancer cells. GADPH was used as a loading control. The abundance of ZNF257 bands relative to the corresponding GADPH bands was assessed densitometrically. E. Cell growth curve of WT and three *ZNF251*KD single clone breast cancer cells following treatment with olaparib. The results represent three independent experiments. F. *ZNF251*KD was constructed in HCC7937 *(BRCA1+* or *BRCA1-)* lines and the resistance to olaparib was measured. The results represent three independent experiments. G, H. The effect of olaparib on the growth of wildtype and *ZNF251*KD MDA-MB-436 breast cancer cells xenografts in immune-deficient NOD/SCID/IL-2Rγ (NSG) mice was tested. The results represent three independent experiments.

To identify genes whose deficiency confers drug resistance to the PARPi olaparib, we performed a genome-wide CRISPR genetic screen in MDA-MB-436 cells, a human breast cancer line harboring a *BRCA1* mutation and which is sensitive to PARPi^15^. We used GeCKO CRISPR library which has been demonstrated to be a very efficient tool to screen for mutations that confer resistance to a BRAF inhibitor in a melanoma line^16^. First, we packed the library into lentivirus with optimal titer at a multiplicity of infection (MOI) of 0.3–0.4 and transduced MDA-MB-436 cells. After viral transduction, we treated the MDA-MB-436 breast cancer cells with either 0.3 μM or 1 μM olaparib, an optimal dose chosen based on our preliminary tests (Supplementary Fig. 1A). After 14 days of treatment, we harvested living cells from the olaparib-treated group and extracted genomic DNA for PCR the region containing sgRNAs. Then, we conducted next-generation sequencing (deep sequencing) to identify sgRNAs enriched in olaparib-resistant cells (Supplementary Fig. 1B). For several genes, we found enrichment of multiple sgRNAs, suggesting that deficiency of these genes contributes to olaparib resistance (Fig. 1A, B). Then, we ranked the positive hits by the number of the sgRNAs and enrichment changes per sgRNA. Interestingly, we identified several zinc finger genes as our highest-ranking genes in our screen. *ZNF251* and *ZNF5T8B* are the only two top hits recovered in the screen at two doses. We tested both genes and found that targeting *ZNF251* resulted in stronger resistance to olaparib. Therefore, we chose to pursue *ZNF251* in this study.

To further validate whether deficiency of *ZNF251* confers resistance to olaparib, we used three newly designed sgRNAs to disrupt *ZNF251* in the MDA-MB-436 breast cancer cell line. We transduced cells with lentivirus-carrying sgRNAs specifically for *ZNF251* and performed the T7 Endonuclease I assay five days after transduction to determine the disruption efficiency. We found that the efficiency of gene disruption ranged from 52.3–89% for all sgRNAs tested (Fig. 1C, top panel). Next, we used these cells to test whether disruption of *ZNF251* can confer resistance to olaparib. Consistent with our screening data, we found that *ZNF251*-deficient cells showed marked resistance to treatment with olaparib compared with the parental cells (Fig. 1C bottom panel). Because the CRISPR/Cas9 genome editing system can create a spectrum of insertions/deletions (in/dels) in a cell population, we also isolated three *ZNF251*-deficient single clones, TOPO cloned and sequenced the PCR product encompassing the targeted region of gRNAs. We found that about 50% of the clones contained Cas9-mediated mutations, including deletions and insertions, at or near the sgRNA PAM (Fig. 1D top panel), indicating that the in/dels were all monoallelic mutations. While it is more common for CRISPR to generate biallelic mutations of a gene, it is not rare to generate monoallelic mutations as well. To further confirm the *ZNF251*-deficient clones’ heterozygosity, we performed a western blot analysis to quantify ZNF251 protein levels in the cells. Our results showed a significant reduction of approximately 50% in protein levels compared to the wildtype control cells (Fig. 1D bottom panel). These findings provide strong evidence that the *ZNF251*-deficient clones are indeed heterozygous knockdowns. Throughout the manuscript, we have referred to the mutation caused by *ZNF251* haploinsufficiency as *“ZNF251* knockdown” (ZNF257KD) to accurately describe it.

Next, we tested drug resistance of three independent *ZNF251*KD clones (#1–3) to olaparib. Consistent with the data from the heterogeneous population of CRISPR-mutated cells, all three *ZNF251*KD clones showed resistance to olaparib compared with the parental (WT) cells (Fig. 1E). The IC_50_ of *ZNF251*-knockdown clones to olaparib was between 7.04 and 16.03 μM, whereas the IC_50_ for parental cells was 4.36 μM. Of note, transfection of *ZNF251*KD cells with an ectopic expression plasmid containing wildtype *ZNF251* cDNA completely reversed resistance to olaparib, indicating that *ZNF251* haploinsufficiency caused the resistance to olaparib (Supplementary Fig. 2A, B).

To address the question of whether the resistance is correlated with *BRCA1* mutation, we knocked down *ZNF251* in an isogenic *BRCA1*-wildtype and -mutated HCC1937 human breast cancer cell lines. We found that *ZNF251* knockdown caused olaparib resistance in *BRCA1*-mutated but not *BRCA1*-wildtype breast cancer cells (Fig. 1F).

We subsequently assessed whether *ZNF251*KD caused PARPi resistance *in vivo*. Experimentally, we tested the effect of olaparib on the growth of parental *(ZNF251* wildtype) and *ZNF251*KD3 MDA-MB-436 cell xenografts in immunodeficient NSG mice. First, we injected either 1×10^6^ wildtype or *ZNF251*KD3 cells subcutaneously into the flank of 16 NSG female mice (8 and 8 mice injected with either *ZNF251* WT or *ZNF251*KD3 cells). Of note, tumors were observed in all 16 animals transplanted with MDA-MB-436 cells in ~3–4 weeks. Next, all animals carrying *ZNF251*WT or *ZNF257*KD3 tumors of 50–60 mm^3^ were randomized into two groups (n = 4/group), which were intraperitoneally treated with vehicle or olaparib (10mg/kg daily for four weeks). As expected, the volume and weight of *ZNF251WT* tumors were strongly reduced when compared to vehicle-treated counterparts (Fig. 1G, H). Remarkably, the tumor size and weight of the olaparib-treated *ZNF251*KD3 group was not reduced by the olaparib treatment, consistent with the resistant phenotype (Fig. 1G, H). This shows that *ZNF251*KD breast cancer cells were resistant to olaparib treatment *in vivo*.

### ZNF251 haploinsufficiency confers resistance to multiple PARPis in BRCA1-mutated cells

**Figure 2.**
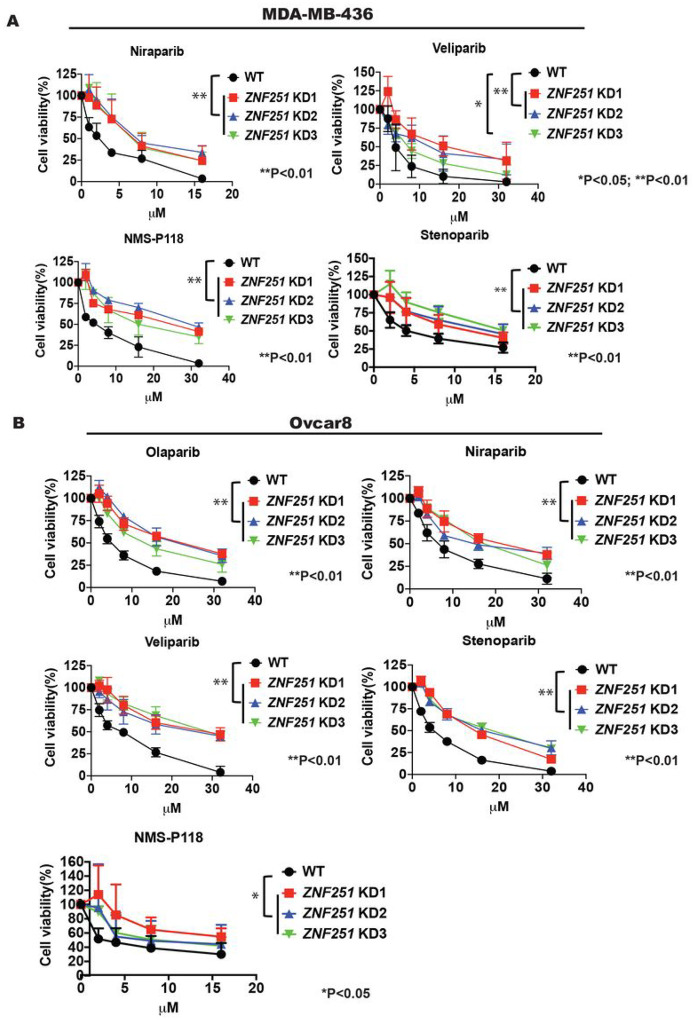
*ZNF251* KD caused resistance to multiple PARP inhibitors in different cancer lines *ZNF251*KD caused resistance to multiple PARPis in different breast and ovarian cancer lines. A, B. *ZNF251*KD was constructed in MDA-MB-436 and Ovcar8 cell lines and the resistance to olaparib, niraparib, veliparib, NMS-P118, stenoparib was measured compared to wildtype (WT) control. The results represent three independent experiments.

To test whether knockdown of *ZNF251* in breast cancer cells induces resistance to additional PARPis, we tested the resistance of *ZNF251*KD MDA-MB-436 clones to several potent PARPis, including niraparib (PARP1/2 inhibitor), veliparib (PARP1/2 inhibitor), NMS-P118 (selective PARP1 inhibitor), and stenoparib (PARP1/2 and PARP5a/5b inhibitor). Consistently, we observed that *ZNF251*KD breast cancer cells were resistant to all those PARPis (Fig. 2A).

To confirm our finding in a *BRCA1*-mutated ovarian cancer line, we knocked down *ZNF251* in the Ovcar8 cell line, a human ovarian cancer line with *BRCA1* mutation, and tested their response to multiple PARPis. We found that *ZNF257*-knockdown ovarian cancer cells were also resistant to those PARPis compared with the *ZNF251*-wild type cells (Fig. 2B). Importantly, in the absence of drug treatment, the growth rate of *ZNF251*-KD breast and ovarian cancer cells was indistinguishable from their wildtype parental cells (Supplementary Fig. 3).

To collect more evidence to support our finding, we also performed bioinformatic analysis of previously published PARPi resistance studies and found significantly lower expression of *ZNF251* in two olaparib-resistant breast cancer cell lines (MDA-MB-468 and SUM1315 lines) when compared to their sensitive counterparts (Supplementary Fig. 4A)^13^·^14^, showing that low expression of *ZNF251* is correlated with olaparib resistance. Furthermore, using CellMiner database analysis, we found that *ZNF251* expression is also positively correlated with sensitivity to olaparib, cisplatin, and carboplatin in breast cancer cells (Supplementary Fig. 4B-D). Consistently, low *ZNF251* expression is correlated with worse survival for breast cancer patients^17^ (Supplementary Fig. 4E). Taken together, downregulation of *ZNF251* was associated with resistance to olaparib and/or platinum derivatives in breast and/or ovarian *BRCA1*-mutated cancer cells. Moreover, cohorts of acute myeloid leukemias (AMLs) display low levels of *ZNF251* (Supplementary Fig. 4F) which may affect the outcome of clinical trials with PARPis^18^.

### ZNF251 haploinsufficiency confers resistance to platinum-based drugs in BRCA1-mutated cells

**Figure 3.**
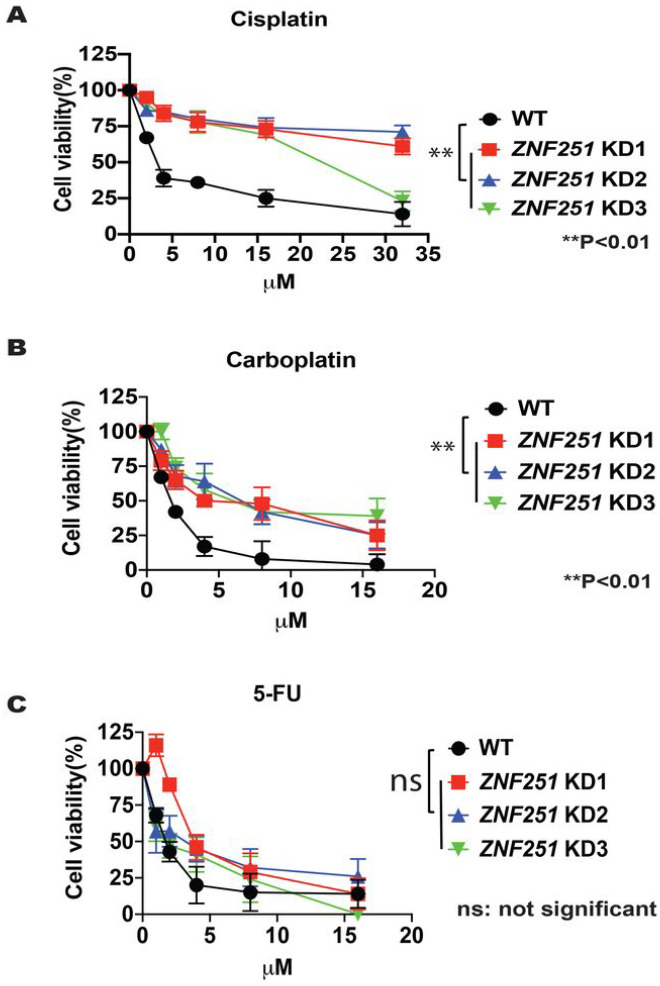
*ZNF251* KD led to resistance to the platinum-based drugs *ZNF251*KD led to resistance to the platinum-based drugs. A-C, the resistance of WT and ZNF251 KDs to cisplatin, carboplatin, 5-fluorouracil was tested. The results represent three independent experiments.

Platinum-based anticancer drugs - including cisplatin, carboplatin, oxaliplatin, nedaplatin, and lobaplatin — are also commonly used first-line chemotherapy regimens in cancer treatment. Mechanistically, these drugs form highly reactive platinum complexes that bind and crosslink DNA in the cancer cells. The mechanisms of action of platinum-based drug and PARP are complementary in many ways and critically reliant on the intracellular DNA damage^19^. It was reported previously that resistance to PARPis also resulted in platinum-based drug resistance^20–21^. Therefore, we tested whether *ZNF251*KD breast cancer cells were resistant to platinum-based drugs. Experimentally, we treated *ZNF251*KD MDA-MB-436 clones with two platinum-based drugs - cisplatin and carboplatin, respectively - and tested drug resistance. As we expected, *ZNF251*KD MDA-MB-436 cells were resistant to both cisplatin and carboplatin (Fig. 3A, B). The IC_50_ of *ZNF251*KD breast cancer clones to cisplatin were 12.54–22.35 μM, whereas the IC_50_ for *ZNF251*WT cells was 1.93 μM. This indicates that *ZNF251* haploinsufficiency confers resistance to platinum-based drugs in *BRCA1*-mutated breast cancer cells. Intriguingly, *ZNF251*KD *BRCA1*-mutated cells were not resistant to 5-fluorouracil (5-FU), which is primarily a thymidylate synthase (TS) inhibitor (Fig. 3C).

### ZNF251 haploinsufficiency confers resistance to DNA polymerase theta (Polθ) inhibitors in BRCA 1-mutated cells

**Figure 4.**
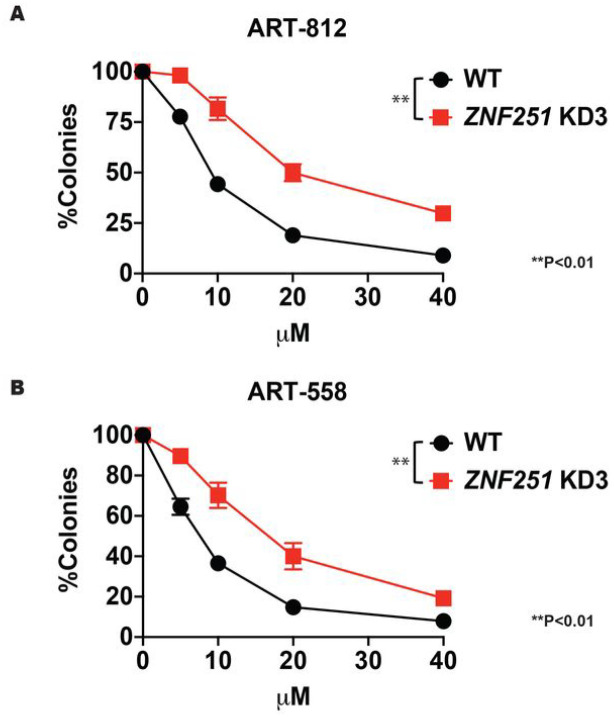
*ZNF251* KD cells showed resistance to Polymerasee inhibitors *ZNF251*KD cells show resistance to DNA polymeraseθ inhibitors. Sensitivity of MDA-MB-436 WT and MDA-MB-436 *ZNF251*KD cells to DNA polymerase θ inhibitors A. ART-812 and B. ART-558 at indicated concentrations. The results represent mean *%* colonies ± SDs when compared to untreated cells.

Recent studies have suggested that HR-deficient cancer cells are sensitive to Polθ inhibitors due to synthetic lethality^22,23^. Moreover, HR-deficient cells resistant to PARPi could be sensitive to DNA Polθ inhibitors^22,23^. To test whether *BRCA1*-mutated *ZNF251*KD breast cancer cells are sensitive to DNA Polθ inhibitors, we treated MDA-MB-436 WT and *ZNF251*KD3 cells with Polθ polymerase inhibitors ART-558 and ART-812^23^ followed by the clonogenic assay. Interestingly, *BRCA1*-mutated *ZNF251*KD3 cells showed resistance to Polθ inhibitors when compared to *BRCA1*-mutated *ZNF251*WT cells (Fig. 4A, B). These results suggest that the *ZNF251* haploinsufficiency confers the resistance to Polθ and PARP inhibitors in BRCA1-mutated cells.

### ZNF251 haploinsufficiency increases NHEJ repair

**Figure 5.**
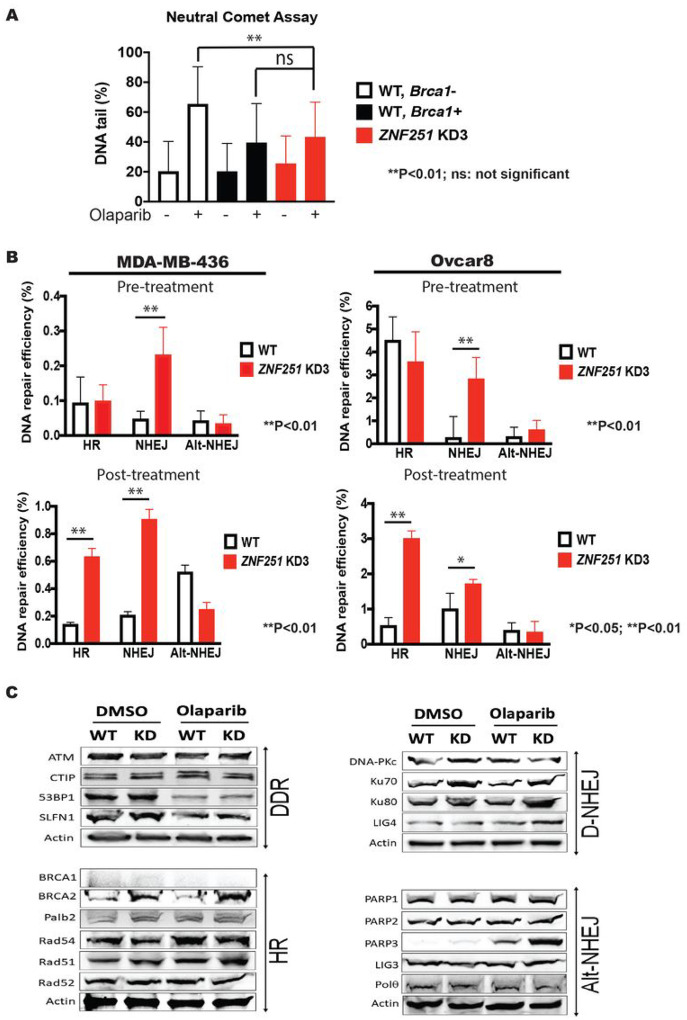
*ZNF251* KD led to upregulated HR and NHEJ repair with treatment of Olaparib *ZNF251*KD resulted in upregulation of HR and D-NHEJ activities. A. Comet assay was performed to examine whether *ZNF251*KD affects the amount of DSBs in MDA-MB-436 cells. B. Reporter assay was carried out to determine the change of DSB repair pathways in MDA-MB-436 and Ovcar8 cells. C. Representative western blots to examine the expression of key components of DSB repair, including HR, D-NHEJ, and Alt-NHEJ.

To determine the molecular mechanisms by which *ZNF251*KD confers drug resistance to PARPi, we first examined DSBs by neutral comet assay in *BRCA1*-mutated wildtype and *ZNF251*KD MDA-MB-436 cells treated with olaparib. We found that *ZNF251*KD MDA-MB-436 cells accumulated less olaparib-induced DSBs when compared to wildtype counterparts and were similar to those detected in BRCA1-restored cells (Fig. 5A), which suggests a restoration of DSB repair in *ZNF251*KD cells.

Therefore, we next examined whether *ZNF251* haploinsufficiency affects DSB repair. Three specific reporter cassettes measuring homologous recombination (HR), non-homologous end joining (D-NHEJ), and alternative non-homologous end joining (Alt-NHEJ) repair activities were applied as described before^11^. Remarkably, we found that NHEJ was markedly upregulated in *ZNF251*KD MDA-MB-436 and Ovcar8 cells before and after the treatment with olaparib, while HR was activated only after olaparib treatment when compared to the wildtype control (Fig. 5B). Alt-NHEJ was not upregulated in *ZNF251*KD MDA-MB-436 cells.

To evaluate specific alterations in DSB repair pathways associated with *ZNF251* haploinsufficiency, RNA-seq was performed and revealed no alterations of the expression of genes involved in D-NHEJ, HR and Alt-NHEJ in *ZNF251*KD cells (Supplementary Table S2). We also performed western blot analysis to examine the expression of the proteins responsible for D-NHEJ, HR and Alt-NHEJ. We found that Ku70 and Ku80 - which are the key components of the canonical DNA-PKcs-dependent D-NHEJ - were upregulated in *ZNF251*KD cells treated or not with olaparib (Fig. 5C, Supplementary Fig. 5). This observation supports the enhanced D-NHEJ activity detected in untreated and olaparib-treated *ZNF251* KD cells. Furthermore, we observed an increased expression of RAD51 - the key elements of the HR pathway in olaparib-treated *ZNF251*KD MDA-MB-436 cells (Fig. 5C, Supplementary Fig. 5) - consistent with stimulation of HR in olaparib-treated *ZNF251*KD cells (Fig. 5B). Altogether, these findings clearly suggest that *ZNF251*KD cells may employ D-NHEJ and eventually also HR to repair olaparib-triggered DSBs to confer the PARPi resistance.

### ZNF251 haploinsufficiency increases DNA-PKcs-dependent replication fork protection in olaparib-treated BRCA7-mutated cells

**Figure 6.**
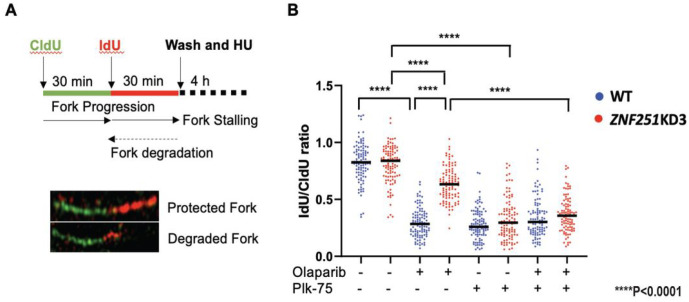
DNA-PKcs inhibition increase the fork degradation of olaparib-treated *ZNF251* KD cell DNA-PKcs inhibitor increased fork degradation in ZNF257KD cells. A Top: schematic representation of the protection of nascent DNA at stalled replication forks employing DNA fiber assay. Bottom: representative images of protected and degraded DNA fibers. B. Graph summarizing the quantification of ldU/CIdU ratio for n = 100 DNA fibers analyzed per sample for each experiment (Cells were treated either 5 μM olaparib and/or 8 μM Plk-75). The graph is representative of 2 independently performed experiments. Significance was calculated with the Mann-Whitney *U*-test, and bar indicated the median for each sample. ****P < 0.0001 differences between samples.

It has been reported that resistance to PARPis induced synthetic lethality might result not only from enhanced DSB repair, but also from enhanced fork stabilization^24^. Intriguingly, DNA-PKcs, independently of its role in D-NHEJ, promoted resistance to PARPi which was associated with fork protection (fork slowing and reversal)^25^. Thus, to further explore the molecular mechanism underlying *ZNF251* haploinsufficiency-caused olaparib resistance, we test whether DNA-PKcs inhibitor (PIK-75) affected stabilization of DNA replication fork. Experimentally, we performed DNA replication fork protection assay (Fig. 6A) in olaparib and/or PIK-75 treated wildtype and *ZNF251*KD cells. As expected, we found that olaparib treatment caused abundant DNA replication fork degradation in MDA-MB-436 wildtype cells whereas only modest effect was observed in *ZNF251*KD (Fig. 6B). This result suggests that *ZNF251* haploinsufficiency protects replication fork from olaparib-induced degradation in *BRCA1*-mutated cells. Remarkably, treatment with DNA-PKcs inhibitor PIK-75 abrogated fork protection in *ZNF251*KD *BRCA1*-mutated cells in the absence and presence of olaparib. These results suggest that in addition to stimulation of DNA-PKcs-mediated D-NHEJ, *ZNF251* haploinsufficiency might activate DNA-PKcs-mediated fork protection to cause resistance to PARPis in *BRCA1*-mutated cells.

### BRCA 7-mutated ZNF257 haploinsufficient cells are sensitive to DNA-PKcs inhibitors

Reactivation of HR pathway was usually reported to cause resistance to PARPis^20^. However, treating *ZNF251*KD MDA-MB-436 cancer cells with RAD57 inhibitor B02 did not reverse the olaparib resistance (Supplementary Fig.S6A, B). Thus, stimulation of HR pathway in olaparib-treated *ZNF251*KD *BRCA1*-mutated breast cancer cells did not play a key role in the resistance.

**Figure 7.**
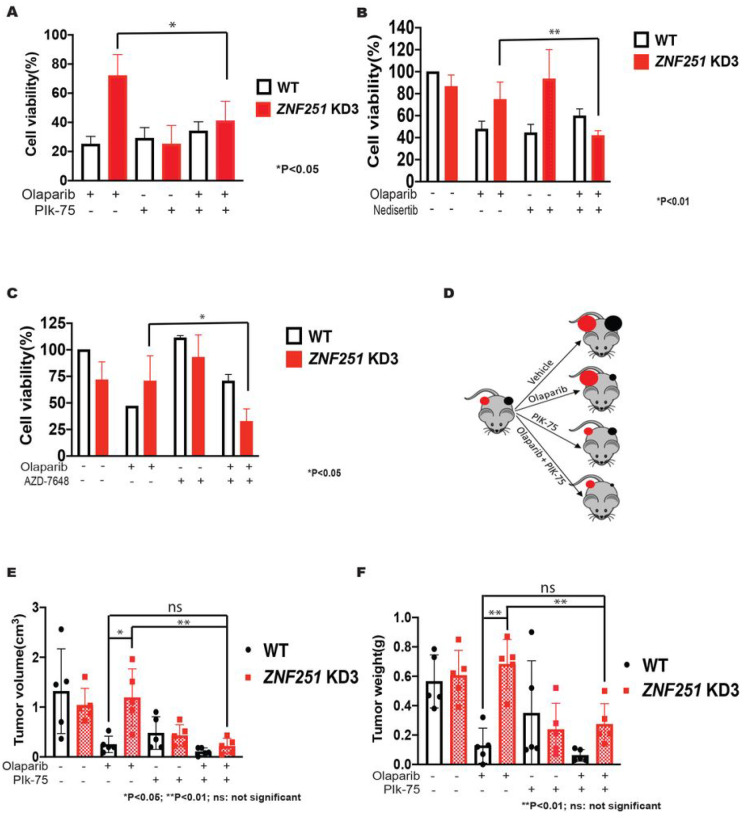
Inhibition of D-NHEJ pathway reversed PARPi resistance in BRCA1-mutated *ZNF251* KD cells Blockade of D-NHEJ pathway reversed PARPi resistance of *ZNF251*KD *in vitro* and *in vivo*. A. Wildtype (WT) and *ZNF251*KD MDA-MB-436 cells were treated with olaparib and DNA-PKcs inhibitor PIK-75 (8 nM). B. Wildtype (WT) and *ZNF251*KD MDA-MB-436 cells were treated with olaparib and DNA-PKcs inhibitor nedisertib (8 μM). C. Wildtype (WT) and *ZNF251*KD MDA-MB-436 cells were treated with olaparib and DNA-PKcs inhibitor AZD-7648 (4 μM). D. Schematic description of our in *vivo* experimental design. E, F. The effect of olaparib and olaparib+PIK-75 on the growth of *ZNF251* wildtype and *ZNF251*KD MDA-MB-436 breast cancer cells xenografts in NSG mice was tested. Results represent mean tumor volume and weight ± SDs.

To test whether constitutively enhanced activity of the D-NHEJ pathway in *ZNF251*KD contributes to the PARPi resistance, we treated *ZNF251*KD MDA-MB-436 cells with olaparib and/or PIK-75, a DNA-PKcs inhibitor. Remarkably, we found that PIK-75 treatment reversed olaparib resistance of *ZNF251*KD MDA-MB-436 cells (Fig. 7A), suggesting that stimulation of DNA-PKcs-mediated D-NHEJ and/or fork protection in *ZNF251*KD cells contributed to the olaparib resistance. Furthermore, we also tested two more DNA-PKcs inhibitors nedisertib and AZD-7648 which are in clinical trials for various cancers^26,27^. Consistently, we found that both DNA-PKcs inhibitors reversed olaparib resistance of *ZNF251*KD MDA-MB-436 cells (Fig. 7B and C).

To evaluate the potential of using DNA-PKcsi to treat olaparib-resistant *BRCA1*-mutated *ZNF251*KD cells in vivo, we conducted an experiment where we treated tumor-bearing mice with PIK-75 and/or olaparib. Specifically, we subcutaneously implanted 1×10^6^ olaparib-resistant *ZNF251*KD cells and olaparib-sensitive *ZNF251* wildtype MDA-MB-436 cells bilaterally to the right and left flank of each NSG mouse, respectively (as shown in Fig. 7D). It is worth noting that tumors were observed in all 20 animals transplanted with wildtype and *ZNF251*KD MDA-MB-436 cells in approximately 3–4 weeks. Once the tumors reached a size of 50–60 mm^3^, we randomly assigned all animals into four groups (n = 5/group) and administered treatment via intraperitoneal injection. The groups received either vehicle, olaparib (10mg/kg), PIK-75 (10mg/kg), or a combination of olaparib and PIK-75. Tumor volume and weight were measured 28 days post-treatment.

As expected, olaparib treatment diminished *ZNF251* wildtype MDA-MB-436 tumor volume and weight by 81 % and 77%, respectively, while *ZNF251*KD MDA-MB-436 tumors were completely resistant (Fig. 7E, F). Remarkably, PIK-75 reduced *ZNF251*KD MDA-MB-436 tumor volume and weight by 64% and 65%, respectively. Moreover, PIK-75 exerted similar antitumor effect against *ZNF251* wildtype MDA-MB-436 tumors. Addition of olaparib to the treatment, did not change the effect of PIK-75. This clearly demonstrates that DNA-PKcsi exerted therapeutic effect against PARPi-resistant *ZNF251*KD MDA-MB-436 breast cancer cells *in vivo*.

## Discussion

Four main mechanisms of acquired PARPi resistance have been identified in *BRCA12*-mutated cancer cells: alteration of drug availability, modulation of de-PARylation enzymes, restoration of HR, and enhanced replication fork stability^7,28^. Using a positive whole-genome CRISPR/Cas9 library screen, and several *BRCA1*-mutated breast and ovarian cancer cell lines we discovered that haploinsufficiency of *ZNF251* which belongs to the Kruppel-associated box (KRAB) zinc-finger gene family cluster caused resistance to multiple PARPis. Mechanistically, we discovered that *ZNF251* knockdown-triggered PARPi resistance was associated with stimulation of two functions of DNA-PKcs: D-NHEJ-mediated DSB repair and D-NHEJ-independent protection of replication forks. Further research is required to determine which process is mainly responsible for the resistance to PARP inhibitors caused by *ZNF251* haploinsufficiency.

ZNFs have been reported to regulate DNA damage response, including DSB repair^29,30^. For example, ZNFs were capable to stimulate (E4F1, ZNF506, ZNF384) and repress (ZNF280C) DSB repair mechanisms such as DNA-PKcs-mediated NHEJ and HR^30–33^. On the other hand, it has been reported that replication fork stability confers PARP inhibitor resistance^20,34^. Our data from *ZNF251*KD cells is consistent with that and mechanistically in concordance with the finding that DNA-PKcs activity is required for this effect^25^. Although we cannot completely rule out the contribution of olaparib-induced activation of HR pathway, RAD51 inhibitor treatment did not reverse the resistance suggested that HR did not play a critical role in PARPi resistance in BRCA7mut + *ZNF251*KD cells. The lack of BRCA1 is most likely compensated by downregulation of 53BP1 and the presence of CtIP in olaparib-treated cells causing imbalance between CtIP-53BP1 (favoring end-resection and thus generating substrates for HR)^35^. In addition, stimulation of HR has been detected only in olaparib-treated *ZNF251*KD cells displaying enhanced expression of RAD51.

Importantly, we showed that *BRCA1*mut + *ZNF251*KD cells were sensitive to a DNA-PKcs inhibitor *in vitro* and *in vivo*, suggesting a novel therapeutic solution. Thus, alterations in ZNFs expression may represent a novel diagnostic tool to pre-screen patients with *BRCA1/2*-mutated tumors for potential treatment with DNA-PKcs inhibitors. However, the detailed molecular mechanism of ZNFs’ function in PARPi resistance still warrants further investigation. Understanding the role of *ZNF251* haploinsufficiency in PARPi resistance can provide insights into drug resistance mechanisms and potential therapeutic strategies.

## Significance Statement

Our study identified a novel gene *ZNF251* which haploinsufficiency causes the resistance to PARP inhibitors in breast cancer cells and pinpointed a novel mechanism of the resistance.
